# Extracellular miR-6723-5p could serve as a biomarker of limbal epithelial stem/progenitor cell population

**DOI:** 10.1186/s40364-022-00384-2

**Published:** 2022-05-31

**Authors:** M. Ruiz, S. González, C. Bonnet, S. X. Deng

**Affiliations:** 1 Cornea Division, Stein Eye Institute, University of California, 100 Stein Plaza, Los Angeles, CA 90095 USA; 2 Cornea Department, Paris University, Cochin Hospital, AP-HP, F-75014 Paris, France

**Keywords:** Limbal stem cells, miRNAs, Biomarker, Cell therapy, Limbal stem cell deficiency, Cornea, miR-6723-5p, Explants culture, Limbal epithelium, Potency assay

## Abstract

**Background:**

Dysfunction or loss of limbal stem cells can result in limbal stem cell deficiency (LSCD), a disease that cause corneal opacity, pain, and loss of vision. Cultivated limbal epithelial transplantation (CLET) can be used to restore stem cell niche homeostasis and replenish the progenitor pool. Transplantation has been reported with high success rate, but there is an unmet need of prognostic markers that correlate with clinical outcomes. To date, the progenitor content in the graft is the only parameter that has been retrospectively linked to success.

**Methods:**

In this study, we investigate extracellular micro RNAs (miRNAs) associated with stem/progenitor cells in cultivated limbal epithelial cells (cLECs). Using micro RNA sequencing and linear regression modelling, we identify a miRNA signature in cultures containing high proportion of stem/progenitor cells. We then develop a robust RNA extraction workflow from culture media to confirm a positive miRNA correlation with stem/progenitor cell proportion.

**Results:**

miR-6723-5p is associated with cultures containing high proportion of stem/progenitor cells, and is detected in the basal layer of corneal epithelium.

**Conclusions:**

These results indicate that miR-6723-5p could potentially serve as a stem/progenitor cell marker in cLECs.

**Supplementary Information:**

The online version contains supplementary material available at 10.1186/s40364-022-00384-2.

## Introduction

The ocular surface is the interface between the eye and the outside world. It is primarily composed of conjunctival epithelium, corneoscleral limbus, and corneal epithelium. Its integrity is ensured by self-renewing cells located at the limbus called limbal stem/progenitor cells (LSCs) [1]. Any damage to these cells or their niche can result in LSC loss or dysfunction, leading to a corneal disease clinically defined as limbal stem cell deficiency (LSCD) [2]. Whereas ocular surface optimization can be sufficient to treat partial LSCD, severe and total LSCD often require surgical treatment [3]. Both direct limbal transplantation and cultivated limbal epithelial transplantation (CLET) can replenish the progenitor pool and/or restore stem cell niche homeostasis [4–6]. Although both LSC transplantation approaches have been reported with high success rate, there is an unmet need of prognostic markers that correlate with clinical outcome. Retrospectively, grafts containing a minimum of 3% of Δp63α^bright^ cells have been associated with a high success rate [7]. Unfortunately, Δp63α is a transcription factor that is not specific to LSCs [8]. Additionally, its detection implies immunostaining, which is a destructive method that requires cells. Therefore, the identification of reliable LSC markers, which quantification does not require disturbing the cells within the graft, is a desirable approach [9].

Micro RNAs (miRNAs), a class of non-coding small RNAs (19–24 nucleotides length) discovered in 1993 in *C. elegans* [10], are known to post-transcriptionally regulate gene expression, and are highly conserved across species [11]. miRNAs bind complementary mRNAs to induce their degradation and/or interfere with translational complexes, resulting in altered protein production [12]. Interestingly, certain miRNAs exhibit a high stability in the extracellular space. This is due to their association with Argonaute proteins [13], along with their packaging within extracellular vesicles released by the cells [14]. Several studies have focused on extracellular miRNAs as potential biomarkers of diseases in serum and plasma [15]. Extracellular miRNAs constitute a reliable tool due to their accessibility, high specificity, and sensitivity. Due to their potential as a biomarkers, miRNAs have been used to differentiate cancer stages and even therapy responsiveness [16]. Similarly, miRNAs can be used to diagnose infectious diseases [17], neurological disorders such as Alzheimer’s disease [18] and cardiovascular diseases [19]. One study has linked the expression of several extracellular miRNAs with induce pluripotent stem cell (iPSCs) differentiation status [20]. In the limbus, several intracellular miRNAs are differentially expressed compared to the central cornea [21]. Extracellular miRNAs in cLEC media could represent accessible molecules that reflect cell status and activity. However, there is no data regarding LSC specific extracellular miRNAs.

In this present study, we aimed to identify extracellular miRNAs that are associated with stem/progenitor cells in cLECs.

## Materials and methods

### Human sclerocorneal tissue and LEC culture

Sclerocorneal tissues (corneoscleral rims) from 18 healthy human donors (20 to 70 year old) were obtained from several eye banks (CorneaGen Inc., Lions Eye Bank, Eversight, Saving Sight, and Vision Share). The study was performed in accordance with the Declaration of Helsinki and the consent for use in research was obtained by the eye banks. The study was exempted by the University of California Los Angeles Institutional Review Board (IRB#12–000363). The death to preservation (DTP) time was less than 12 hours and the death to experiment (DTE) time was less than 7 days. Tissues were conserved in Optisol-GS storage media until dissection. For miRNAscope experiment, donors were less than 50 years old with a DTP time less than 7 hours (h), a death to tissue processing time less than 3 days, and none or minimal epithelium sloughing.

Prior to isolating LECs, the iris, endothelium, conjunctiva, and Tenon’s capsule were removed. Two by two millimeters limbal explants dissected from corneoscleral rim were seeded with the epithelium facing up on amniotic membrane (AM) from selected C-section procedures after thermolysin denudation, as previously described [22]. Limbal explants were cultivated in a modified version of SHEM media [23]. In brief, modified SHEM (mSHEM) consisted of the DMEM/F-12 medium (ThermoFisher Scientific) supplemented with 5% human serum (Access Biological, LLC), 1% CTS N2 supplement (ThermoFisher Scientific), 1% penicillin-streptomycin (Hyclone GE Healthcare), 0.4 ng/mL of epidermal growth factor (R&D Systems), 1 μg/mL of isoproterenol (Nexus Pharmaceuticals), 0.5 μg/mL of hydrocortisone (Sigma Aldrich Corp.), 0.01 mg/mL gentamicin (ThermoFisher Scientific), and 0.25 μg/mL amphotericin B (Hyclone GE Healthcare). Medium was replaced every 48 h.

### Conditioned media processing

**Fig. 1:**
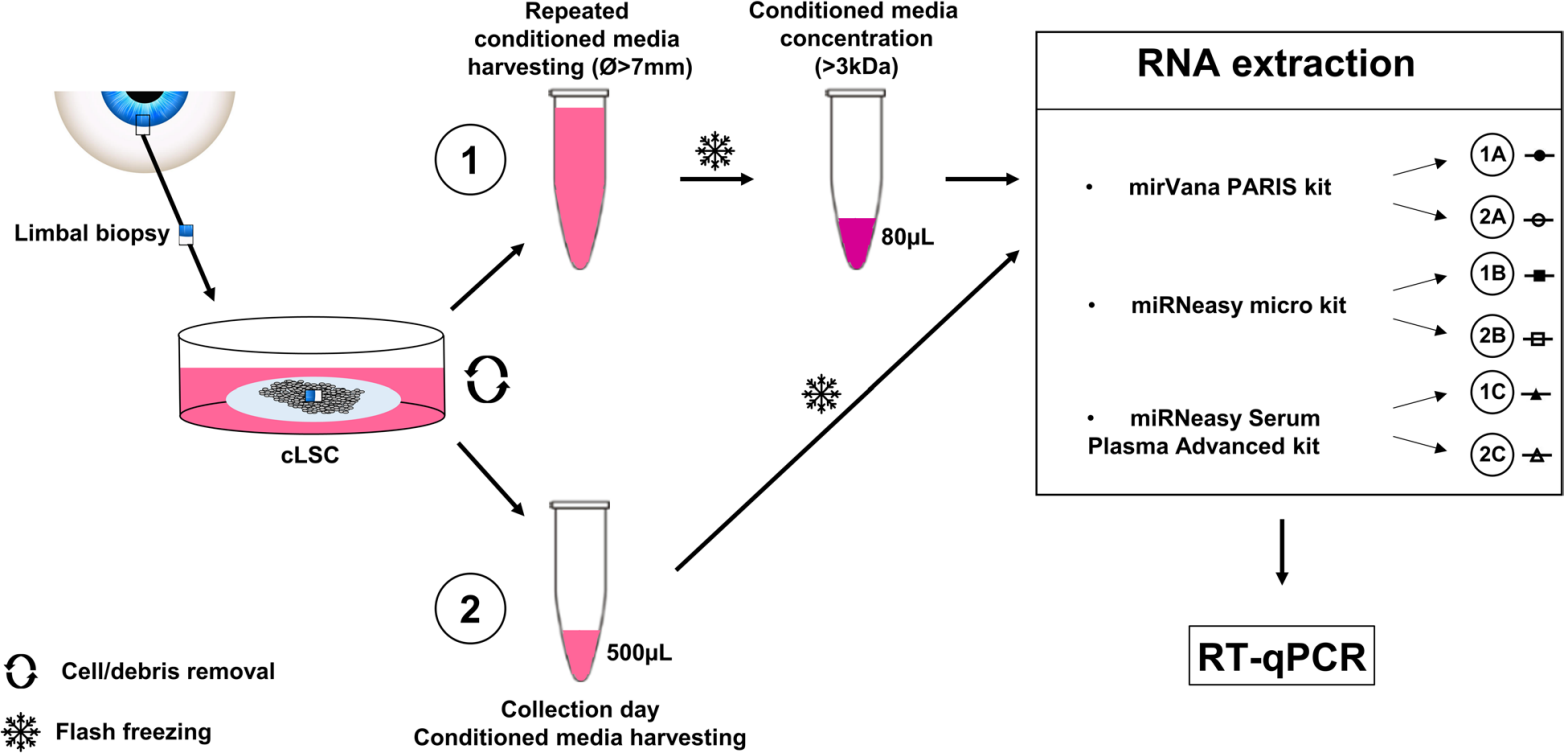
Workflow of RNA extraction from cLEC culture media. Overview of the methodology developed to select the RNA extraction method from cLEC conditioned media. In arm 1, media is harvested every 48 hours once the average diameter of the explant outgrowth reached 7 mm. Cells and debris were removed before flash freezing. Media was concentrated (~ 22 times) using a 3 kDa exclusion column prior to RNA extraction. In arm 2, media is harvested at a unique time point corresponding to cell collection. Centrifugation allows for cell and debris removal before flash freezing. Subsequently, media is processed for RNA extraction. In both arms, total volume is divided by three and processed with three different miRNA extraction kits. RNA quality and concentration are determined using both Nanodrop and Bioanalyzer small RNA electrophoresis. A, B and C refer to mirVana PARIS, miRNeasy micro and miRNeasy Serum/Plasma Advanced kits respectively. 1 and 2 refer to the concentrated and unconcentrated arm respectively

Briefly, conditioned media were harvested every 48 h until cLEC collection. Supernatants were centrifugated 10 min at 500G followed by 15′ at 2000G (both at 4 °C) to remove cells and debris. The supernatant was flash frozen in liquid nitrogen and stored at − 80 °C. In arm 1 (See Fig. 1), 5 mL of pooled conditioned media from different time points were concentrated approximately 22 times by centrifugation. This was done a using 3 kDa Ultra-15 centrifugal filter units (Millipore) for 90′ at 4000G at 4 °C. In arm 2, unconcentrated media from collection day were directly used for RNA extraction.

### Analysis of cell morphology, cell density, cell size, and immunohistochemistry study

Prior to cLEC collection, images of proximal and peripheral areas were taken on the corneal, conjunctival, and both limbal sides (8 pictures/cLEC) with inverted light microscopy. Cell morphology was graded and cell density was calculated using ImageJ software (NIH).

cLEC collection was performed as previously described [23]. Briefly, cLECs were incubated in 2.4 U/mL of dispase II (Roche) in DMEM/F-12 (ThermoFisher Scientific) at 37 °C for 2 h. The cell sheet was detached from AM by gentle successive pipetting over the edges. A single cell suspension was obtained by incubating with 0.25% trypsin-EDTA (Life Technologies) for 5 min at 37 °C. Single cell suspension was placed in a hemocytometer, and pictures were captured using the BZ-X710 microscope (Keyence). The percentage of cells with a diameter smaller than 12 μm was determined using BZ-X analyzer software (Keyence). cLECs that did not present an epithelial morphology, had less than 50 cells/40,000 μm^2^, or less than 1% of small cells were excluded from the study.

Immunocytochemistry was performed as previously described [23], with the antibodies listed in Supplementary Table 1. Image analysis of the cell size and immunostainings were done using BZ-X analyzer software (Keyence) and the hybrid cell count function. By specifying a mask area according to several parameters, the software extracted information such as cell counts, target area measurements, and fluorescent signal in different channels. Individual masked images were confirmed by human eye. When necessary, the masking parameters were fine tune in order to detect all the cells and deliver accurate proportions. The software analyzed a minimum of 500 cells/sample. Cells expressing high levels of ΔP63α (noted as ΔP63α^bright^ cells) were quantified following the previously described method [24]. To note, commercially available antibodies against p63α lack specificity and recognize both p63α isoforms: TAp63α and ΔP63α. Since ΔP63α is abundant in limbus [25, 26], a pattern of high level of expression in cLECs is considered to mainly represent ΔP63α expression.

### HTG EdgeSeq miRNA whole transcriptome

Eighteen concentrated cLEC conditioned media (35 μL) pooled from different time points were submitted for analysis using HTG EdgeSeq miRNA whole transcriptome assay (HTG Molecular Diagnostics, Tucson, AZ, USA). The volume of media and HTG lysis buffer were mixed (1/1 ratio) to obtain working concentration. To improve sample lysis, proteinase K was added (1/10th of lysis buffer volume) and samples were incubated at 50 °C for 180 min. Twenty-five microliters of each sample was added to a single well of a 96-well plate. Human brain RNA (25 ng) was also added by HTG staff in triplicate to serve as an internal control.

Samples were run on an HTG EdgeSeq processor using the HTG EdgeSeq human miRNA whole transcriptome panel of 2083 miRNAs. The HTG EdgeSeq Parser was used to align the FASTQ files to the probe list and data was reported on an excel file as raw, Quality Controlled (QC) raw, Count per Million (CPM), and median normalized read counts.

### RNA extraction and RT-qPCR

Due to small conditioned media input volumes, we investigated the efficiency and repeatability of 3 different RNA extraction kits: miRVana PARIS kit (ThermoFisher Scientific), miRNeasy micro kit (Qiagen), and miRNeasy Serum/Plasma Advanced kit (Qiagen). Concentrated and unconcentrated approaches were assessed in parallel as described in Fig. 1. One hundred femtomol of synthetic cel-miR-39-3p mimic (ThermoFisher Scientific) were spiked in each sample, and cLEC media input volumes were standardized.

A modified version of the miRVana PARIS kit (ThermoFisher Scientific) for biofluid samples was used as previously described [27]. For miRNeasy micro kit (Qiagen), cLEC conditioned media was thawed by adding a 5:1 ratio of QIAzol lysis reagent. Chloroform was added at a 1:1 ratio in the subsequent step. Thereafter, the protocol was followed as per manufacturer’s instructions. For miRNeasy Serum/Plasma Advanced kit (Qiagen), the protocol was followed as instructed by the manufacturer. RNA concentration was assessed using the Bioanalyzer small RNA chip (Agilent) and the Nanodrop (ThermoFisher Scientific) using the wavelength dependent extinction coefficient “33”. miRNA extraction from cells was performed as per manufacturer’s instructions using miRNeasy micro kit (Qiagen).

RT-qPCR was used to validate miRNA candidates identified by sequencing. One nanogram (ng) for cel-miR-39-3p, or 10 ng for all other miRNA candidates were used for reverse transcription (RT) with TaqMan microRNA RT kit (ThermoFisher Scientific). Ct values from three technical replicates were averaged, and targets of interest normalized to cel-miR-39-3p. Results are presented as relative expression using the formula 2^-ΔCt^.

### miRNA in situ hybridization using miRNAscope assay

The miRNAscope HD Assay Red (ACD Biosystems, Newark, CA) was performed on fresh-frozen tissue as per manufacturer’s instructions with the following protocol adjustments. A 1 h baking at 60 °C was performed before fixation in 4% PFA for 50 min. H_2_O_2_ incubation time was increased to 20 min, and a 20 min post-fixation step in 4% PFA was performed after protease treatment. Hybridization time was decreased to 90 min. Twelve micrometers (μm) corneal sections from three donors were hybridized with the custom designed probe against miR-6723-5p. Small RNA integrity and signal specificity were confirmed with a positive control probe targeting human small nucleolar RNA RNU6B, and a negative control probe targeting a miR-6723-5p scramble sequence. Fluorescent images were obtained with the BZ-X710 fluorescence microscope (Keyence).

### Statistical analysis

For sequencing data, technical variation of read counts for each sample was removed using three different normalization strategies: log2 (CPM), median-ratio, and quantile. A linear regression model was fitted with quantile normalized miRNA expression as the response and the percentage of ΔP63α^bright^ cells as the predictor for all non-control probes with average raw expression greater than 30 reads. Additionally, four control media were included as covariates to account for varying levels of background miRNAs from the different lots of human serum used in media composition. Raw *p*-values were used to interpret results. A second analysis approach was carried out using DESeq2 package (version 1.14.1) available from Bioconductor [28]. Samples were segregated into two groups: low (< 4%) and high (> 15%) ΔP63α^bright^ cells.

Additional statistical test information is indicated in the legend of each figure. One, two or three symbols indicate a *p* value < 0.05, < 0.01 and < 0.001, respectively.

## Results

### cLECs are heterogenous

**Fig. 2:**
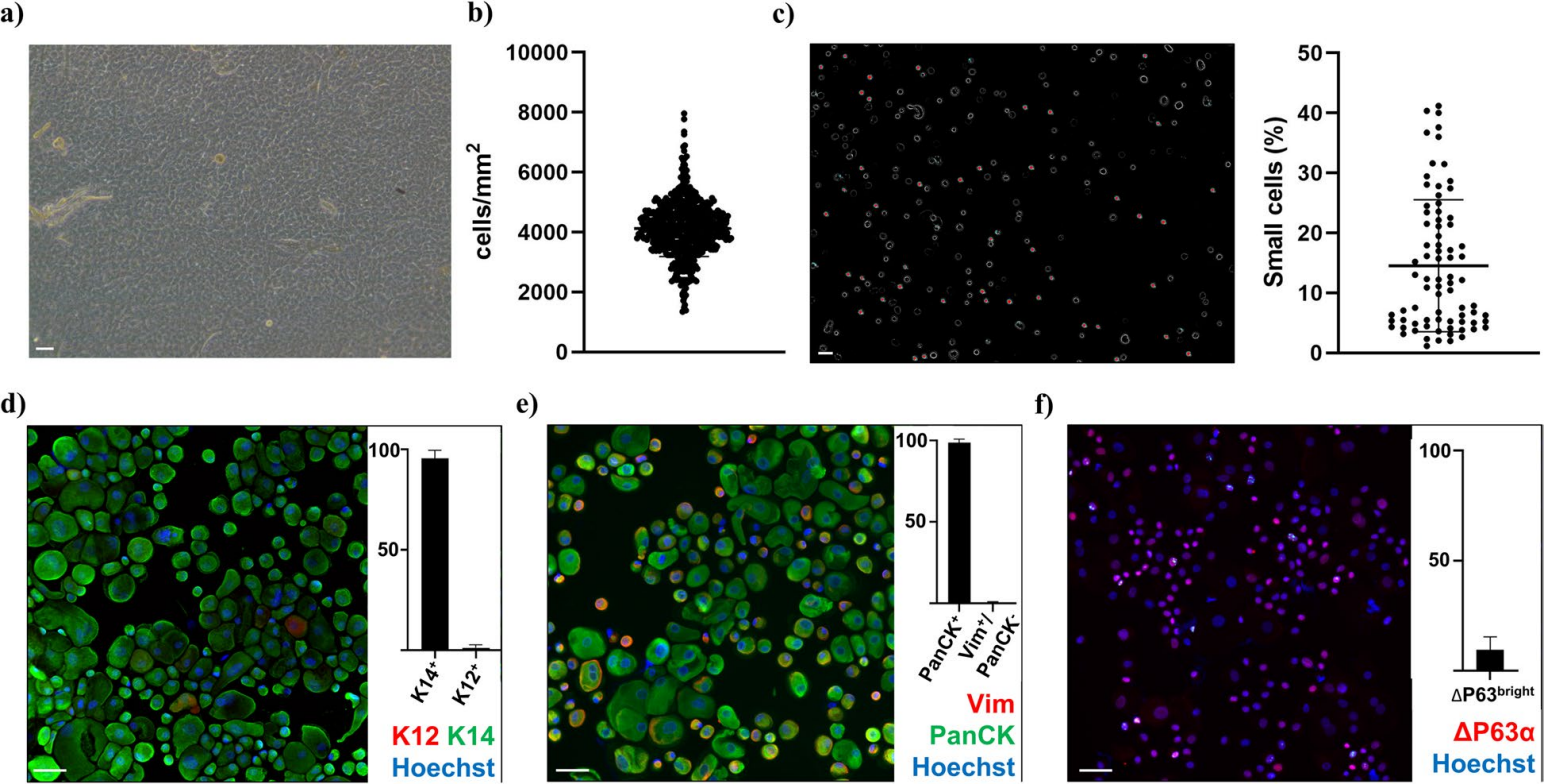
cLEC characterization and phenotyping. **a** Representative picture of the epithelial morphology observed in cLECs as seen by contrast phase microscopy (10X). **b** Average cell density obtained from the 624 graded areas of 40, 000μm^2^ (8 areas/cLEC). **c** Software detection and quantification of small cells with diameter inferior to 12 μm (10X). **d** Phenotypic characterization and quantification of cLSCs using immunofluorescent labelling of K12 (red), K14 (green) and Hoechst dye (blue); **e** Vimentin (red), PanCytokeratin (green) and Hoechst dye (blue); **f** ΔP63α (red) and Hoechst dye (blue). Representative pictures as seen by fluorescent microscopy (20X). Scale bar is set at 50 μm

A total of 72 cLECs from 12 different corneal tissue donors were included. Cultivation and phenotyping parameters are listed in Table 1. The average cultivation time was 9.54 days (ranged from 8 to 11 days), and the average outgrowth diameter was 19.4 ± 2.0 mm. A typical epithelial-like morphology was observed in 99.66% ± 2.12% of the graded areas (Fig. 2a). The average cell density was 4119 ± 934 cells/mm^2^ (Fig. 2b). There was an average of 14.52% ± 10.92% of small cells (ranged from 1.14 to 41.19%) (Fig. 2c).

**Table 1:** cLSC features and phenotype

	Average	SD	Range
**Days in culture**	**9.54**	**1.12**	**8–11**
**Outgrowth diameter (mm)**	**19.4**	**2**	**6–20**
**Epithelial-like morphology (%)**	**99.66**	**2.12**	**87.5–100**
**Cell density (cells/mm**^**2**^**)**	**4119**	**934**	**1342–7960**
**Small cells**	**14.52**	**10.92**	**1.14–41.19**
**K14**^**+**^ **(%)**	**95.58**	**3.96**	**79.34–99.82**
**K12**^**+**^ **(%)**	**1.22**	**1.51**	**0–7.32**
**PanCK**^**+**^ **(%)**	**98.53**	**2.19**	**86.67–100**
**PanCK−/Vim**^**+**^ **(%)**	**0.35**	**0.64**	**0–3.73**
**ΔP63αbright (%)**	**9.43**	**5.91**	**1.66–33.1**

To further characterize cLECs, we analyzed the expression of the progenitor cell markers Δp63α and K14, along with the differentiated cell marker K12. The percentage of K14^+^ cells was 95.58% ± 3.96%, while K12^+^ cells represented only 1.22% ± 1.51%, indicating a majority of undifferentiated cells (Fig. 2d). We also confirmed the absence of major stromal contamination by analyzing the double staining for a common epitope of epithelial cytokeratins: pancytokeratin (PanCK), along with the intermediate filament vimentin expressed mostly by stromal cells [29]. The percentage of PanCK^+^ cells was 98.53% ± 2.19%, while PanCK^−^/Vim^+^ was only 0.35% ± 0.64% (Fig. 2e). The percentage of Δp63α^bright^ cells ranged from 1.66 to 33.1%, with an average of 9.43% ± 5.91% (Fig. 2f). There was no correlation between the number of cells collected and the percentage of Δp63α^bright^ cells (data not shown). Since the percentage of ΔP63α^bright^ cells is the only parameter that has been correlated with clinical success [7], we used this criterion to select conditioned media samples for sequencing.

### Extracellular miRNA profiles differ in conditioned media of cLECs with different proportions of ΔP63α^bright^ cells

**Fig. 3:**
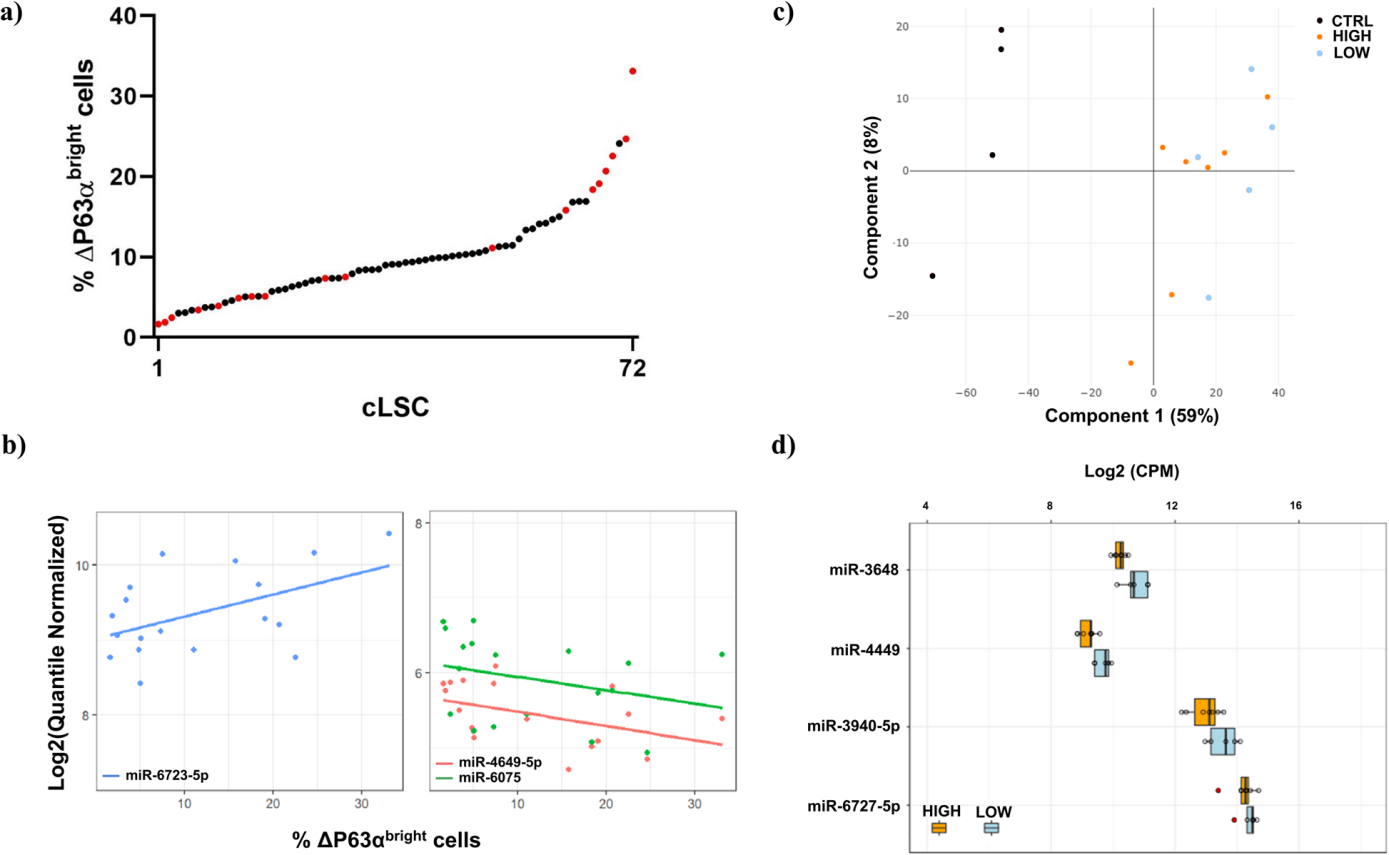
Linear modeling and differential expression of cLEC extracellular miRNAs in response to varying ΔP63α^bright^ cell percentages. **a** cLEC ΔP63α^bright^ cell percentage distribution across 72 cLECs. Red dots represent the 18 samples selected for HTG EdgeSeq miRNA whole transcriptome assay. **b** Plot distribution of quantile transformed miRNA counts against ΔP63α^bright^ cell percentages. Linear trend for the top significantly regulated targets miR-6723-5p (left panel), miR-6075 (right panel - green) and miR-4649-5p (right panel - red). **c** PCA plot of the miRNA data characterizing the trends exhibited by the expression profiles of ΔP63α^bright^ LOW (blue) and HIGH (orange) cLEC conditioned media samples. Unconditioned CTRL media are depicted in black. **d** Top 4 most differentially expressed miRNAs in cLEC conditioned media from LOW and HIGH ΔP63α^bright^ cell groups

Based on the percentage of ΔP63α^bright^ cells, we selected 18 samples from the 72 cLEC conditioned media library for the HTG EdgeSeq miRNA whole transcriptome sequencing. This approach was selected because miRNA extraction was not necessary. The range of percentage of ΔP63α^bright^ cells is depicted in Fig. 3a. Log2 (CPM), median-ratio, and quantile normalization strategies were assessed (Supplementary Figure 1-a). The latter was used to fit a linear regression model with “miRNA expression” as the response, and “percentage of ΔP63α^bright^ cells” as the predictor. A total of three miRNAs showed significant slope. The linear trend for all probes modelled is shown in Supplementary Figure 1-b, and the 30 most significant miRNAs identified are represented in Supplementary Figure 1-c. miR-6723-5p showed a significant positive correlation (*p* = 0.018) with a predicted fold change of 2.53 between the lowest and highest ΔP63α^bright^ cell percentage (Supplementary Table 2). miR-4649-5p and miR-6075 showed a significant negative correlation (*p* = 0.024 and *p* = 0.047 respectively) with predicted fold changes of − 1.84 and − 1.43. Additional control sequencing runs with pools containing an identical input of conditioned and control media were assessed to control background miRNA levels. We confirmed that the miRNA signal was originated from the media conditioned by the cells, rather than the human serum contained in the unconditioned control media itself (Supplementary Table 3). miR-4649-5p in 11 samples, and miR-6075 in 3 samples did not reach the 30 reads detection threshold, indicating a low detection confidence. Therefore, both miRNAs were excluded from the subsequent analysis. When performing a sensitivity analysis to determine the effect of quantile normalization on linear regression modelling, miR6723-5p was the only miRNA significantly correlated with the percentage of ΔP63α^bright^ cells in all three data transformation strategies (cut-off: *p*-value< 0.05) (Supplementary Figure 1-d and Supplementary Table 4).

Since linear regression modelling is sensitive to inter-donor variability, we also used a different strategy to identify cLSC specific extracellular miRNAs. cLECs were segregated into LOW (< 4%) and HIGH (> 15%) ΔP63α^bright^ cell groups, and DESeq2 differential gene expression analysis was performed. The Principal Component Analysis (PCA) segregated the unconditioned control media from the cLEC conditioned media samples (Fig. 3c). No clear segregation was observed between the control media and cLEC conditioned media, indicating that the majority of expressed miRNAs were shared between cLECs with different proportions of ΔP63α^bright^ cells. DESeq2 statistical analysis revealed that four targets were significantly upregulated in the LOW group: miR-3648 (*p* = 0.0299, FC = -1.76), miR-4449 (p = 0.0299, FC = -1.72), miR-3940-5p (*p* = 0.0497, FC = -1.82), and miR-6727-5p (p = 0.0497, FC = -1.50) (Fig. 3d). miR-6727-5p was excluded as the fold change was less than 1.5.

### Association of miR-6723-5p with high percentage of ΔP63α^bright^ cells in LECs cultures

**Fig. 4:**
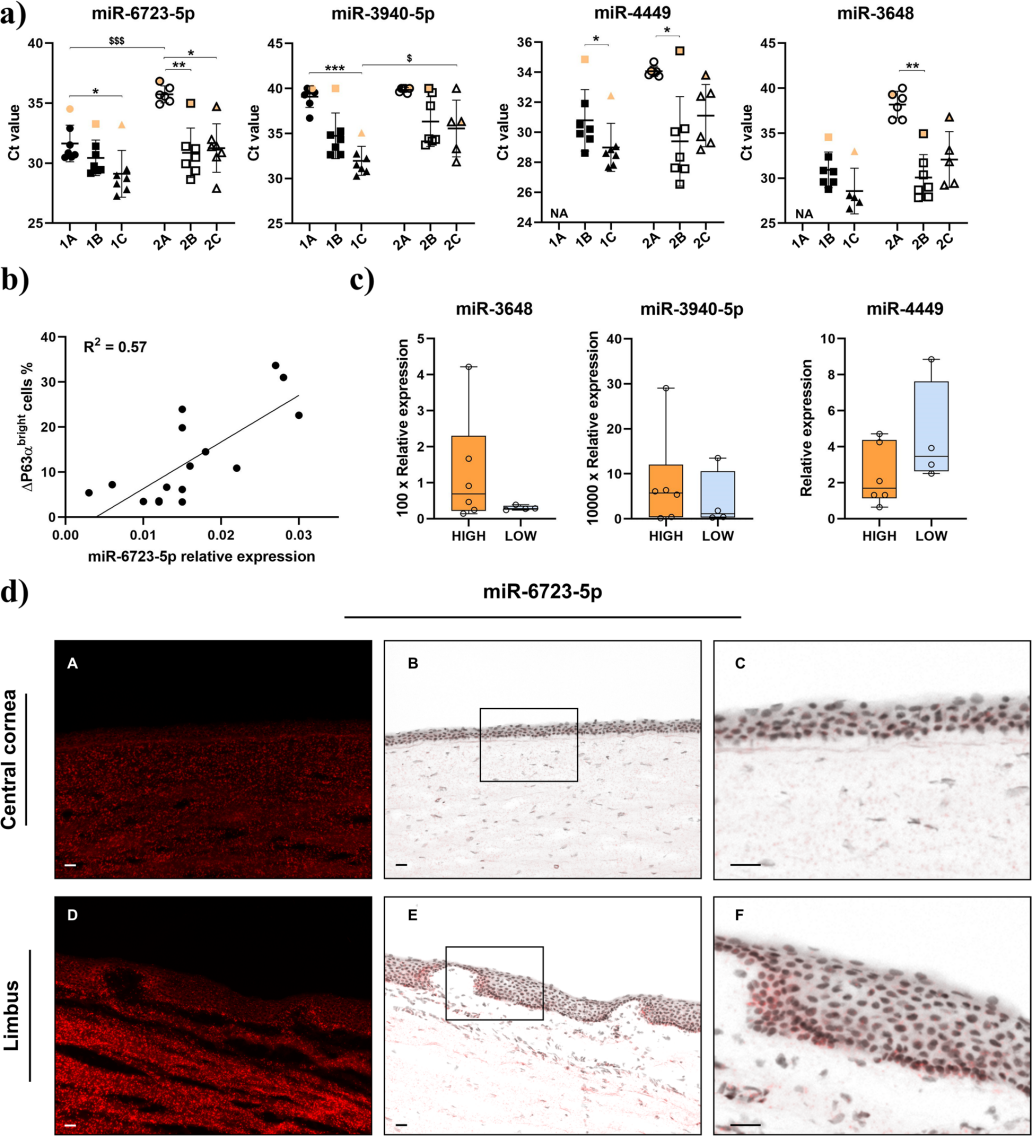
miRNA targets validation. **a** Ct values obtained for the amplification of miR-6723-5p, miR-3648, miR3940-5p, and miR-4449 by RT-qPCR using the RNA extraction workflow described in Fig. 1. Control media is depicted in orange color. Multiple comparisons tested with *: Kruskal-Wallis or $: 2 way ANOVA tests respectively. NA: Not Available due to low RNA recovery efficiency in arm 1A. **b** Plotted values of miR-6723-5p relative expression against ΔP63α^bright^ cell percentage. R^2^ indicates the Pearson correlation value between the two variables. **c** Relative expression of miR-3648, miR-3940-5p and miR-4449 in samples from two datasets classified in HIGH (orange) and LOW (blue) groups. Relative expression of miR-3648 and miR-3940-5p have been multiplied by 100 and 10,000 respectively to facilitate representation. **d** Representative fluorescent staining of miR-6723-5p (red) in both central cornea (A,B) and limbus (D,E) using the miRNAscope assay. Panels C and F correspond to higher magnification of squared areas in panels B and E respectively. Scale bar is set at 50 μm

Due to the small sample volume, to ensure the efficiency and repeatability of RNA isolation from culture media, three different RNA extraction kits: miRVana PARIS, miRNeasy micro and miRNeasy Advanced Serum/Plasma were compared as described in Fig. 1. A spike RNA was used as a control for isolation efficiency. Spike recovery and RNA yield results are depicted in Supplementary Figure 2a and b, c respectively. miR-6723-5p, miR-3940-5p, miR-4449, and miR-3648 were also quantified (Fig. 4a). Similar trends in Ct values were obtained in all isolation methods. In most cases, the highest Ct values were found to be in the control media. Contrary to the trend observed for RNA yields, the detection of miRNAs happened earlier in the concentrated arm 1 (Fig. 1), which is in line with the increased conditioned media input. Ultimately, Advanced Serum/Plasma used with concentrated media consistently provided the earliest detection for all miRNA targets, along with a consistent segregation of the control media. Additionally, the recovery of the spike miRNA reached 70.19% on average (data not shown). This method was therefore selected for targets validation.

In order to confirm the findings from HTG EdgeSeq miRNA whole transcriptome sequencing, we next quantified the four miRNA targets prospectively using the Advanced Serum/Plasma RNA isolation method in 17 cLEC conditioned media. Consistent with the linear regression model fitted for sequencing data analysis, miR-6723-5p expression was positively correlated with the percentage of ΔP63α^bright^ cells (*p* = 0.0005) (Fig. 4b). Pearson correlation coefficient was 0.75, and 57 % of the variance was shared between miR-6723-5p expression and the percentage of ΔP63α^bright^ cells.

In parallel, we quantified the expression levels of miR-3648, miR-3940-5p and miR-4449 identified by DEseq2 analysis (Fig. 4c). There were no significant differences between HIGH and LOW ΔP63α^bright^ cells group samples. miR-4449 exhibited an upregulation trend in the LOW ΔP63α^bright^ cell group, but did not reach statistical significance (*p* = 0.26).

### Confirmation of miR-6723-5p in human limbal basal epithelium

To further correlate miR-6723-5p with progenitor cells, RNAscope was performed to locate this miRNA in human eye tissue. Interestingly, the miR-6723-5p expression was mainly detected in the basal region of the limbal epithelium, where LSCs are located. In contrast, miR-6723-5p was not detected in the superficial layer of limbal epithelium and the central cornea (Fig. 4d and Supplementary Figure 3).

## Discussion

Transplantation of cultivated limbal epithelial cells could successfully restore LSC function in eyes with LSCD [5]. To date, the success of LSC transplantation has only been retrospectively associated with the proportion of ΔP63α^bright^ progenitor/stem cells within the graft [7]. Characterization of cLECs prior to transplantation without sacrificing cultivated cells is of paramount importance for assessing the quality of the graft during expansion, and optimizing clinical success. The current study shows that extracellular miRNAs could be isolated from culture media, and that the level of miR-6723-5p in the culture media correlates with the amount of ΔP63α^bright^ progenitor/stem cells.

miRNAs from cell conditioned culture media have shown a predictive value in systems such as in vitro fertilization, with a 96.6% similarity of the miRNA signature between trophectoderm cells and their conditioned media [30]. Comparably, changes in miRNA expression pattern during iPSC generation were correlated with their culture media content [20], suggesting that extracellular miRNAs could be used to determine cell phenotype and monitor stemness status. On the ocular surface, miRNAs play an important role. miR-31, miR-145, miR-146a and miR-184 regulate LSC differentiation and/or play a role in corneal epithelial homeostasis [31–36]. miR-10b and miR-184 have been identified in the limbus and central cornea, respectively [37], and several miRNAs are dysregulated in diabetic cornea [21, 38]. More recently, small RNA sequencing identified miRNA profiles in enriched corneal epithelial stem cells and mature central corneal epithelial cells [39]. Altogether, these findings suggest that miRNA signatures are associated with specific cell types or diseases.

Identification and quantification of extracellular miRNA are challenging due to the low quantity and high noise from the media. Additionally, the process requires several steps, which are often time consuming and can lead to biased interpretation. Sample preparation, RNA extraction, reverse transcription, RT-qPCR, and normalization strategies can all impact results [40, 41]. Standardized protocols are essential to ensure reproducibility and enable the comparison of results across different experiments, before the protocols can be used in clinical setting. miRNeasy Serum/Plasma Advanced kit appears to achieve a higher yield of RNA isolation compared to the other two RNA isolation methods tested. Concentration of media does not improve miRNA yield, but is associated with a higher reproducibility among samples. Using Advanced Serum/Plasma kit, we successfully detect miR-6723-5p in cLEC cultured media by RT-qPCR, and confirm its upregulation in cultures containing a higher percentage of ΔP63α^bright^ cell prospectively.

The absence of consensus on endogenous controls is a tremendous challenge for the use of RT-qPCR in miRNA quantification, especially in the context of extracellular miRNAs [42]. No universally invariant calibrator or other small RNA have been identified in conditioned media, neither in any biofluids. RNU6B, usually used to normalize miRNA in qPCR [13], was either heterogeneously detected or absent in cLEC conditioned media samples (data not shown). To overcome this challenge, we used a synthetic cel-miR-39-3p to normalize RT-qPCR data. This method allowed us to monitor the robustness of RNA isolation process. Absolute quantification could also represent a good alternative once miRNA targets are identified [43].

HTG EdgeSeq has shown good correlation with qPCR and dPCR when performed on human plasma specimens [44]. Reproducible results have also been obtained with sample inputs as low as 20 μL [45]. Although heterogeneity between cLEC conditioned media was high, linear modelling revealed a significant correlation between miR-6723-5p content in the conditioned media and the percentage of ΔP63α^bright^ cells in the cLECs. miR-6723-5p has only been described in two recent studies. Its expression is upregulated by Homeobox B5, a poor prognosis marker in pancreatic cancer [46]. Using RNA-seq, the authors showed that miR-6723-5p upregulation is associated with embryonic stem cell pathways, along with an increase in cell proliferation and migration. miR-6723-5p-mimic promotes proliferation in colony formation assay of human pancreatic adenocarcinoma cell line, as well as cell migration in wound healing assays. miR-6723-5p is also upregulated in xenograft model of hypopharyngeal tumor treated with ^188^Re-liposome [47]. Its increased detection in conditioned media of cLECs containing high percentage of ΔP63α^bright^ cells might reflect an undifferentiated state and a higher proliferative capacity, which are features of LSCs in culture. Consistent with this finding, we detected miR-6723-5p in the basal layer of the limbal epithelium where LSCs are located [48]. Both ΔP63α and Frizzled-7, another putative LSC marker, are mostly localized in the limbal basal layer. Further colocalization study is necessary to confirm if miR-6723-5p is also colocalized with ΔP63α and Frizzled-7.

It is still unclear if miRNA concentration is systematically increased by the higher sample volume. miRNAs could have different expression kinetics throughout the course of LSC expansion in culture, and pooling media during the entire course of culture could result in miRNA dilution rather than concentration. Similarly, the kinetic of ΔP63α^bright^ cell content during cultivation is unknown. In clinical settings, release criteria for cellular therapy products are often required to strictly reflect the product to be transplanted. By combining media from different harvest time points, our approach rather represents a potency assay.

There are a couple of limitations in the current study. First is the relatively small sampling size. A prospective study with a larger sample size is necessary to confirm our finding. Second, the analysis was restricted to a specific panel of 2083 miRNAs, and the possibility that other miRNAs might have specific regulation pattern should not be excluded.

In conclusion, a robust method has been developed to assess miRNAs in culture media. miR-6723-5p is correlated with limbal epithelial stem/progenitor cells in vitro and in vivo. miR-6723-5p could serve as a biomarker of stem/progenitor cell content in cLECs.

## Supplementary Information

**Additional file 1: Supplementary Figure 1.** HTG Edgeseq miRNA whole transcriptome sequencing data normalization. a) miRNA count distribution using Log2 (CPM), median-ratio and quantile data transformation strategies. b) Linear trend for all probes with count values centered. c) Regression plot for the 30 most significant miRNAs identified in the quantile normalised data. Different shapes represent independent HTG EdgeSeq run while colours refers to the human serum used in cLEC media. Black dotted lines represent the trend for the first run of samples while the solid blue line indicate the trend for both runs (all samples). d) Overlap in significantly regulated miRNAs between linear models fitted to the three data transformation strategies. miR-6723-5p overlaps with all transformation strategies.

**Additional file 2: Supplementary Figure 2.** RNA extraction efficiency from cLEC conditioned media using different methods. a) RT-qPCR amplification of synthetic spike-in cel-miR-39-3p in RNA extracted from 6 cLEC conditioned media and one control media following the workflow described in Fig. 1, 100fmol of cel-miR-39-3p were added in each sample before extraction. b) miRNA concentration (pg/μL) in the 10 to 40 nucleotides region determined with small RNA chip Bioanalyzer and c) with Nanodrop. Multiple comparisons tested with *: Kruskal-Wallis or $: 2 way ANOVA tests respectively.

**Additional file 3: Supplementary Figure 3.** miR-6723-5p staining in human cornea. Localization of miR-6723-5p (red) in both central cornea (K,N) and limbus (L,O) in two additional donors using the miRNAscope assay. Negative control corresponds to a scramble sequence of miR-6723-5p and is depicted in central cornea (A,B) and limbus (C,D). Positive control targets the small-nucleolar RNA RNU6B, and is depicted in central cornea (F,G) and limbus (H,I). Panels E, J, M, and P correspond to higher magnification of squared areas in panels D, I, L, and O respectively. Scale bar is set at 50 μm.

**Additional file 4: Supplementary Table 1.** Antibodies list.

**Additional file 5: Supplementary Table 2.** Linear regression model metrics for significantly regulated probes.

**Additional file 6: Supplementary Table 3.** TOP3 differentially expressed miRNAs read counts.

**Additional file 7: Supplementary Table 4.** Linear regression model metrics for each data normalization strategy.

## Data Availability

NA.

## Abbreviations

AM: Amniotic membrane
CPM: Count per million
CLET: Cultivated limbal epithelial transplantation
cLSCs: Cultivated limbal stem/progenitor cells
cLECs: Cultivated limbal epithelial cells
DTE: Death to experiment
DTP: Death to preservation
h: Hours
iPSCs: Induce pluripotent stem cell
LSCD: Limbal stem cell deficiency
miRNAs: micro RNAs
μm: Micrometers
PanCK: Pancytokeratin
PCA: Principal component analysis
QC: Quality controlled
RT: Reverse transcription
