# Supplementary figures and images for: Extracellular miR-6723-5p could serve as a biomarker of limbal epithelial stem/progenitor cell population
Source: Biomark Res. 2022 May 31;10:36. doi: 10.1186/s40364-022-00384-2 (PMC9153202; doi:10.1186/s40364-022-00384-2)

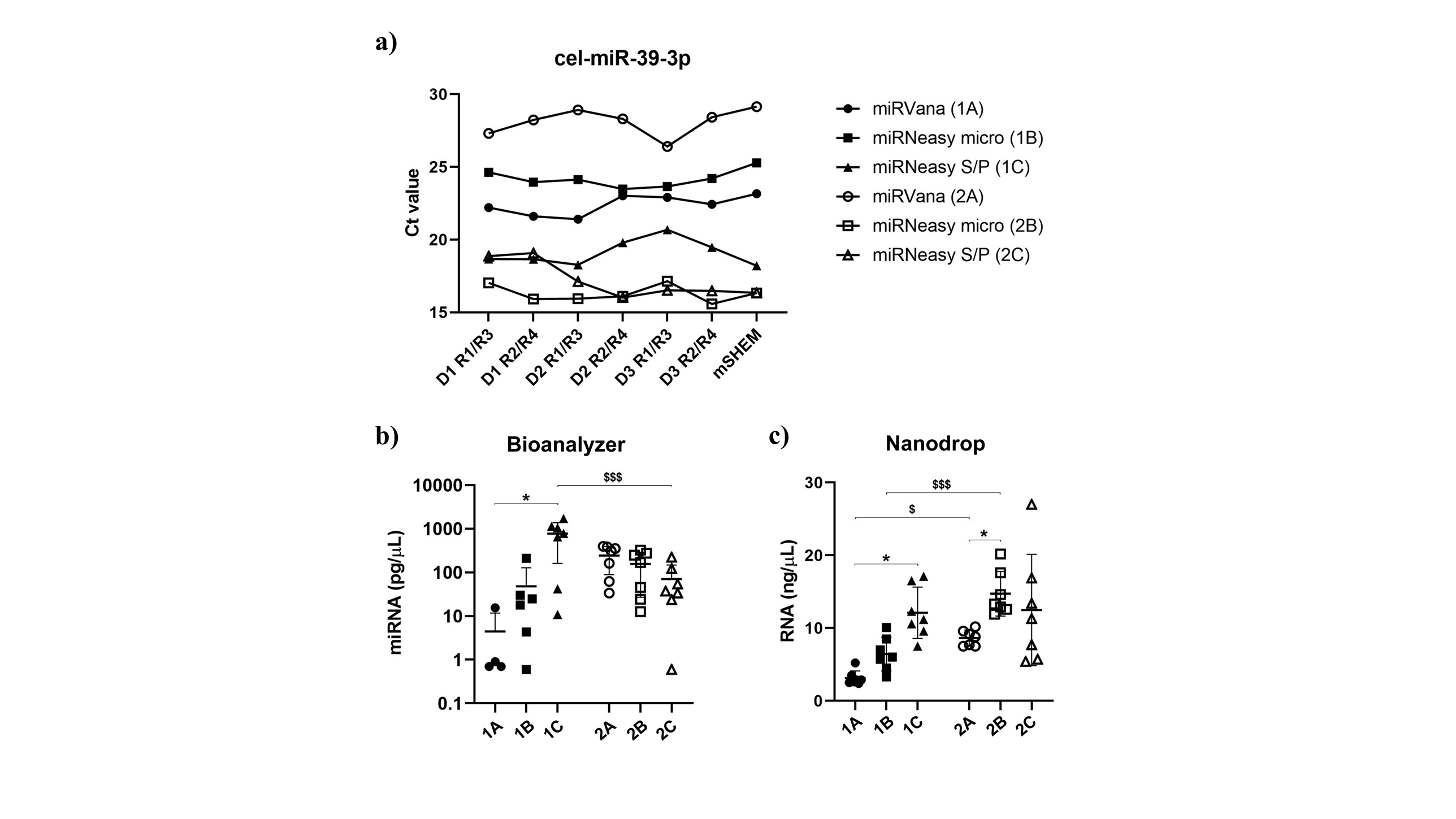

Supplement: Supplementary file 2 — Additional file 2: Supplementary Figure 2. RNA extraction efficiency from cLEC conditioned media using different methods. a) RT-qPCR amplification of synthetic spike-in cel-miR-39-3p in RNA extracted from 6 cLEC conditioned media and one control media following the workflow described in Fig. 1, 100fmol of cel-miR-39-3p were added in each sample before extraction. b) miRNA concentration (pg/μL) in the 10 to 40 nucleotides region determined with small RNA chip Bioanalyzer and c) with Nanodrop. Multiple comparisons tested with *: Kruskal-Wallis or $: 2 way ANOVA tests respectively. [file 40364_2022_384_MOESM2_ESM.tif]
