# Supplementary material for: Extracellular miR-6723-5p could serve as a biomarker of limbal epithelial stem/progenitor cell population
Source: Biomark Res. 2022 May 31;10:36. doi: 10.1186/s40364-022-00384-2 (PMC9153202; doi:10.1186/s40364-022-00384-2)
Supplement: Supplementary file 4 — Additional file 4: Supplementary Table 1. Antibodies list. [file 40364_2022_384_MOESM4_ESM.pdf]

# Supplementary Table 1) Antibodies list

| Antibody target | Source | Catalogue number | [Stock] | Dilution |
|---|---|---|---|---|
| Keratin 12 (K12) | Santa Cruz Biotechnology | sc-25722 | 200µg/mL | 1:100 |
| Keratin 14 (K14) | Fisher Scientific | MS-115-R7 | Not Available | 1:50 |
| Cytokeratins (PanCK) | Dako Omnis | M3515 | Not Available | 1:100 |
| Vimentin (Vim) | Abcam | ab92547 | 1.062mg/mL | 1:100 |
| p63α | Cell Signaling Technology | 4892 | Not Available | 1:100 |
| Alexa Fluor 488 | ThermoFisher Scientific | A11029 | 2mg/mL | 1:500 |
| Alexa Fluor 546 | ThermoFisher Scientific | A11035 | 2mg/mL | 1:500 |
