# Supplementary material for: Extracellular miR-6723-5p could serve as a biomarker of limbal epithelial stem/progenitor cell population
Source: Biomark Res. 2022 May 31;10:36. doi: 10.1186/s40364-022-00384-2 (PMC9153202; doi:10.1186/s40364-022-00384-2)
Supplement: Supplementary file 5 — Additional file 5: Supplementary Table 2. Linear regression model metrics for significantly regulated probes. [file 40364_2022_384_MOESM5_ESM.pdf]

Supplementary Table 2) Linear regression model metrics for significantly regulated probes

Probe	Estimated Slope	P-value	Predicted log(Fold Change)	Predicted Raw Fold Change
miR-6723-5p	0.04	0.01	1.34	2.53
miR-4649-5p	-0.02	0.02	-0.88	-1.84
miR-6075	-0.01	0.04	-0.52	-1.43
