# Supplementary material for: Extracellular miR-6723-5p could serve as a biomarker of limbal epithelial stem/progenitor cell population
Source: Biomark Res. 2022 May 31;10:36. doi: 10.1186/s40364-022-00384-2 (PMC9153202; doi:10.1186/s40364-022-00384-2)
Supplement: Supplementary file 6 — Additional file 6: Supplementary Table 3. TOP3 differentially expressed miRNAs read counts. [file 40364_2022_384_MOESM6_ESM.pdf]

Supplementary Table 2) TOP3 differentially expressed miRNAs read counts

	LOW					HIGH							CTRL			
**$\Delta P63\alpha^{bright}$ (%)**	1.66	1.88	2.45	3.43	3.90	15.80	18.39	19.11	20.68	22.55	24.68	33.10				
**HS media composition**	HS7	HS7	HS3	HS7	HS7	HS7	HS1	HS8	HS8	HS8	HS3	HS8	HS1	HS3	HS7	HS8
**miR-3648**	1454	2545	2600	2070	4524	1085	2988	854	2070	1086	1646	893	147	112	179	134
**miR-4449**	1035	1335	1294	912	2089	520	1568	454	1029	596	883	418	36	30	161	102
**miR-3940-5p**	11477	26663	19765	8064	35548	9930	10666	9128	9339	8393	10246	9482	38	89	15	23
