# Supplementary material for: Extracellular miR-6723-5p could serve as a biomarker of limbal epithelial stem/progenitor cell population
Source: Biomark Res. 2022 May 31;10:36. doi: 10.1186/s40364-022-00384-2 (PMC9153202; doi:10.1186/s40364-022-00384-2)
Supplement: Supplementary file 7 — Additional file 7: Supplementary Table 4. Linear regression model metrics for each data normalization strategy. [file 40364_2022_384_MOESM7_ESM.pdf]

Supplementary Table 4) Linear regression model metrics for each data normalization strategy

Quantile

Probe	Estimated Slope	P-value	Predicted log(Fold Change)	Predicted Raw Fold Change
miR-6723-5p	0.04	0.01	1.34	2.53
miR-4649-5p	-0.02	0.02	-0.88	-1.84
miR-6075	-0.01	0.04	-0.52	-1.43

Median-ratio

Probe	Estimated Slope	P-value	Predicted log(Fold Change)	Predicted Raw Fold Change
miR-6723-5p	0.04	0.03	1.11	2.16
miR-4461	0.03	0.03	1.05	2.07

CPM

Probe	Estimated Slope	P-value	Predicted log(Fold Change)	Predicted Raw Fold Change
miR-4306	0.03	0.00	1.04	2.06
miR-30d-5p	0.02	0.03	0.65	1.57
miR-221-3p	0.02	0.04	0.69	1.61
miR-6723-5p	0.04	0.04	1.28	2.43
miR-4429	0.01	0.04	0.49	1.40
miR-21-5p	0.03	0.04	0.89	1.85
miR-29c-3p	0.03	0.04	0.91	1.88
miR-92b-3p	0.02	0.04	0.70	1.62
miR-4463	0.03	0.04	1.00	2.00
